# Investigating a Work System Approach to Implement an Emergency Department Surge Management System: Case Study

**DOI:** 10.2196/37472

**Published:** 2022-08-25

**Authors:** Jennifer Jewer

**Affiliations:** 1 Faculty of Business Administration Memorial University of Newfoundland St. John's, NL Canada; 2 Cross-appointed, Discipline of Emergency Medicine, Faculty of Medicine Memorial University of Newfoundland St. John's, NL Canada

**Keywords:** emergency department, surge management, work system, system implementation, emergency department information system, mobile phone

## Abstract

**Background:**

Emergency department (ED) crowding is a global health care issue. eHealth systems have the potential to reduce crowding; however, the true benefits are seldom realized because the systems are not integrated into clinicians’ work. We sought a deep understanding of how an eHealth system implementation can be structured to truly integrate the system into the workflow.

**Objective:**

The specific objectives of this study were to examine whether work system theory (WST) is a good approach to structure the implementation of an eHealth system by incorporating the entire work system, and not just the eHealth system, in the implementation framework; identify the role that specific elements of WST’s static framework and dynamic work system life cycle model play in the implementation; and demonstrate how WST can be applied in the health care setting to guide the implementation of an eHealth system.

**Methods:**

Through a case study of an ED in a rural hospital, we used a mixed methods approach to examine the implementation of a surge management system through the lens of WST. We conducted 14 hours of observation in the ED; 20 interviews with clinicians, management, and members of the implementation team; and a survey of 23 clinicians; reviewed related documentation; and analyzed ED data to measure wait times. We used template analysis based on WST to structure our analysis of qualitative data and descriptive statistics for quantitative data.

**Results:**

The surge management system helped to reduce crowding in the ED, staff was satisfied with the implementation, and wait time improvements have been maintained for several years. Although study participants indicated changes to their workflow, 72% (13/18) of survey participants were satisfied with their use of the system, and 82% (14/17) indicated that it was integrated with their workflow. Examining the implementation through the lens of WST enabled us to identify the aspects of the implementation that made it so successful. By applying the WST static framework, we saw how the implementation team incorporated the elements of the ED work system, assessed their alignment, and designed interventions to address areas of misalignment. The dynamic work system life cycle model captured how planned and unplanned changes were managed throughout the iterative implementation cycle—83% (15/18) of participants indicated that there was sufficient management support for the changes and 80% (16/20) indicated the change served an important purpose.

**Conclusions:**

The broad scope and holistic approach of WST is well suited to guide eHealth system implementations as it focuses efforts on the entire work system and not just the IT artifact. We broaden the focus of WST by applying it to the implementation of an ED surge management system. These findings will guide further studies and implementations of eHealth systems using WST.

## Introduction

### Background

Emergency department (ED) crowding is a major global health care issue [1]. The negative consequences are well established and include adverse patient outcomes and increased mortality [1,2]. ED crowding describes a situation in which the demand for emergency services exceeds the ability to provide care in a reasonable amount of time. When an ED has reached the point of overcapacity, the hospital implements a process, called surge, to allow for decompression. The use of eHealth systems, including systems to manage surge, has the potential to offer numerous benefits for EDs; however, the benefits have often been less than anticipated in many cases owing to implementation difficulties [2]. In particular, the difficulty of integrating the system into clinicians’ work is cited as a key barrier to the successful implementation of ED [3] and other eHealth systems [4,5]. Several systematic reviews of eHealth interventions found that workflow was one of the most common barriers to successful implementations [6-8]. Granja et al [6] recommended that there is a critical need to perform in-depth studies of the workflow when implementing eHealth interventions to identify facilitators and barriers at the earliest possible stage of the implementation to ensure that they are defined in the implementation strategy. This highlights the need for those undertaking such projects to understand the factors that affect the staff’s work and workflow, so that they can modify and improve the implementation to align with the organization’s and staff’s requirements. However, we lack a thorough understanding of how the implementation of such systems can be structured to incorporate the broad work system.

### Objectives

We propose that using work system theory (WST) [9,10] to guide the implementation of eHealth systems may help to attain desired outcomes by incorporating the entire work system into the implementation framework. To evaluate whether WST will be a good approach, we examined the implementation of a surge management system in an ED of a rural, Canadian, 80-bed hospital through the lens of WST.

The surge management system was designed to track patient demand and capacity in the ED, calculate surge levels, and prescribe volume-based staffing. One of the most important eHealth systems in the health care domain is the ED information system to manage information and workflow and support patient care in the emergency room, and there are numerous studies supporting the advantages of its use [2]. However, although surge management is often a component of many ED information systems and there are a plethora of studies examining many processes to manage surge (ie, lean management [11], small cycles of process changes [12], and implementation of fast-tracking [13]), there are few studies examining eHealth systems specifically for the management of surge in the ED.

WST is a well-established theory for understanding relationships between technology and work systems. A work system is “a system in which human participants and/or machines perform work (processes and activities) using information, technology, and other resources to produce specific products/services for specific internal and/or external customers” (p75) [9]. WST is based on the premise that systems, and the work processes they affect must be properly managed to fit with practice. The adoptive entity is not only the system but also the entire IT-enabled work system. WST views the work system from two perspectives: (1) a static framework, with (2) a dynamic life cycle. The static framework presents a view of the work system at a particular time interval through 9 elements constituting the work system’s form, function, and environment. According to WST, the 6 internal elements of the static framework—processes and activities, participants, information, technologies, customers, products, and services—should be balanced. The remaining 3 external elements—environment, infrastructure, and strategies—provide the context in which the work system operates. The dynamic work system life cycle (WSLC) model, presents how the work system changes over time through planned and emergent (unplanned) changes. In this study, we used a mixed methods approach to examine the implementation of the surge management system from a WST perspective and conceptualize the eHealth system as only a component within a broad ecosystem. We propose that WST can be useful for structuring the implementation of such eHealth systems.

## Methods

### Overview

We followed a longitudinal case study methodology over approximately 2 years using a mixed methods approach to collect data during and after the implementation of the surge management system. The ED is located in a rural hospital that provides emergency and inpatient services to a catchment population of approximately 40,000. At any point of time, the ED has 8 stretchers, 3 high-turnover examination beds, and average daily volume of approximately 80 patient visits. The ED team is made up of 1 primary family physician who practices emergency medicine, 1 secondary coverage physician, 1 nurse practitioner, and up to 4 registered nurses per shift. In total, the ED has approximately 50 staff and management directly involved in surge management.

### Surge Management System—Background

The surge management system is composed of the eHealth system and related interventions to manage surge levels in the ED (ie, new surge management processes and procedures and new personnel). When we refer to the surge management system in this study, we are not only referring to the eHealth system portion but also to the other components of the new surge management process. This reflects the view of WST that technology is only an element of the work system. The surge management system was implemented iteratively over approximately 5 years, and our study examines the last 2 years of the implementation, and in particular, the implementation of the eHealth system portion of the surge management system. The implementation team did not follow a formal implementation framework; instead, there was a unique situation in which the surge management system was developed by 2 ED clinicians, and they led the implementation.

**Figure 1:**
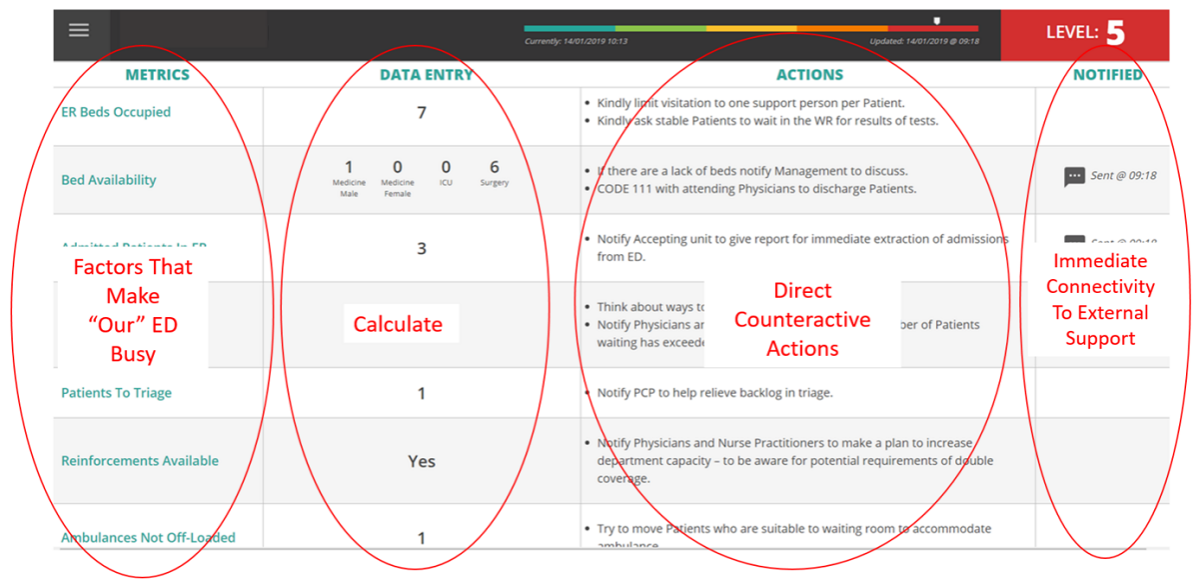
Screenshot of the surge management system.

The eHealth system portion of the surge management system is installed in the ED nursing station, and staff receives notifications of surge levels through a smartphone app via email and SMS text messages (Figure 1). Patient demand and capacity are entered at least every 2 hours for identified metrics that influence waiting times in the ED. Data are entered more frequently if the staff perceives the patient demand to be increasing. A surge level score is calculated in real time using algorithms. Each score has a corresponding set of prescribed volume-based staffing, management, and overcapacity protocols. For example, a total score of 40 triggers the highest level (level 5) and is associated with actions such as sending a text to frontline management and staff, calling ‘Surge Level 5,’ sending all patients with low acuity to the waiting room, and contacting physicians with potential discharges.

### Data Collection and Analysis

**Figure 2:**
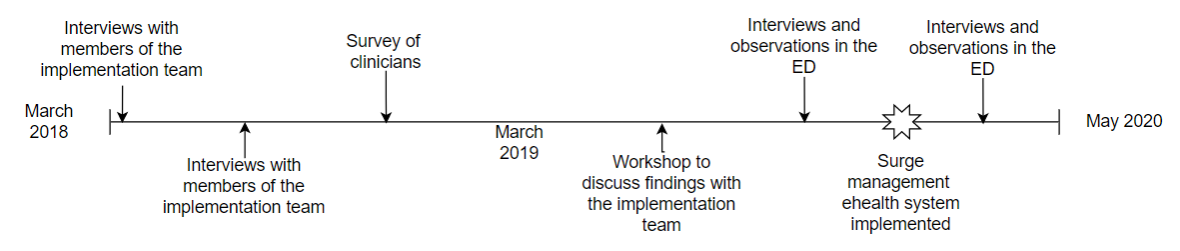
Time line of data collection. ED: emergency department.

We started our primary data collection several months after most of the manual changes to the surge management process were in place and as the team was beginning the implementation of the eHealth system portion. At this time, the clinicians used a manual version of the surge management system, in which they calculated surge levels by hand and manually reported the surge levels. Figure 2 shows a time line of our data collection.

We started by conducting open-ended interviews with 2 key members of the implementation team over a period of several months. These interviewees also worked in the ED and were key participants in the surge management process. This gave us background information about the decision to implement the surge management system, overview of the hospital and ED functioning, and insight into the ongoing and planned implementation processes.

After 4 months, we distributed a web-based survey to the clinical staff in the ED to determine their satisfaction with the surge management system, the perceived benefits to the hospital, and the nature of their job level change. We also asked about their perceptions of fairness of the change, management support for the change, and their commitment to the change. Validated measures were used and adapted for our setting where necessary [14-18]. The survey was completed by 23 clinicians who play a direct role in surge management at the hospital. Though the sample size for the surveys appears small (n=23), it represents approximately 50% of the population of potential users. Refer to Multimedia Appendix 1 for the survey questions. To cope with the statistical limitations of the small survey sample size, we confined our quantitative analyses to descriptive statistics. We conducted a workshop with members of the implementation team to discuss the initial observations and to receive feedback, thereby increasing internal validity [19].

Then, we used the findings from the initial interviews and survey to inform our observations in the ED and to probe areas further through additional interviews approximately 9 months later and again after 4 months once all aspects of the eHealth system implementation were complete. We conducted 14 hours of observation over 2 days (1 day during the implementation process and 1 day after the eHealth portion of the surge management system, and all related changes were implemented). Each day included several clinical shifts so that we could see how the process functioned at various times throughout the day and at various surge levels. We had full access to the ED and our observations involved observing the environment, staff interactions and events, activities, and processes occurring in the hospital, which are associated with the use of the surge management system. We took notes on what was observed and asked questions to understand what was happening. Refer to Multimedia Appendix 1 for the observation protocol. During this time, we also conducted 16 interviews with frontline clinicians and management, and members of the implementation team were included in this group. Refer to Multimedia Appendix 1 for the interview guide. We stopped our phases of observations and interviews when data saturation was reached. All notes and recordings of interviews and observations were transcribed, and all sources were anonymized. We reviewed documentation on the system and the implementation, including documentation on the surge management system and the surge protocol, implementation plans, business case for the surge management system, and specification of outcome measures. Table 1 provides an overview of the data collection.

**Table 1:** Data collection overview.

Data source	Details	Respondent characteristics
Surveys	A total of 23 respondents	A total of 13 internal support (n=4, 31% primary RNs^a^; n=1, 8% triage RN; n=5, 38% primary care paramedics; and n=3, 23% ED^b^ physicians) and 10 external support (n=1, 10% admitting physician; n=3, 30% RN on inpatient unit; and n=6, 60% other)
Observations	A total of 14 hours of observation	Observed the ED over 2 days and several clinical shifts—1 day during the system implementation and 1 day after the implementation. Observed clinicians using and interacting with the system and managing flow in the ED. Full access was available wherever required.
Interviews	A total of 20 interviews	Of the 20 interviews, 4 (20%) were with ED physicians, 11 (55%) were with ED RNs, 2 (10%) were with nurse practitioners, 1 (5%) was with primary care paramedic, 1 (5%) was with ED manager, and 1 (5%) was with patient care facilitator (inpatient beds)
Document review	Surge management system documentation, surge protocol documentation, implementation plans, business case for the surge management system, and specification of outcome measures	N/A^c^
ED wait time data	Patient ED wait times from point of registration to patient departure from the ED, from April 1, 2017, to March 31, 2021	N/A

^a^RN: registered nurse.

^b^ED: emergency department.

^C^N/A: not applicable.

**Figure 3:**
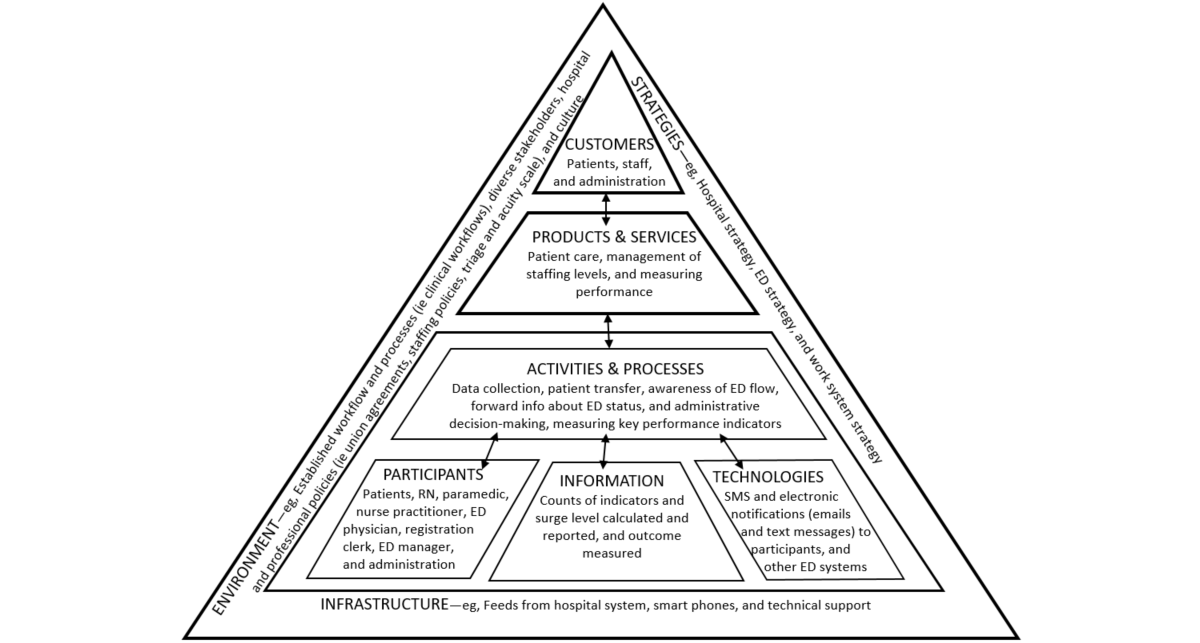
Static framework for the surge management system (adapted from the publication by Alter [9]). ED: emergency department; RN: registered nurse.

We used template analysis [20] based on the elements of WST to structure the qualitative data analysis. Template analysis forced us to take a well-structured approach to handle the data [20] and allowed us to examine the data according to the elements of WST. We started by coding the data according to the static framework. The 9 elements of the static framework adapted to the implementation of the surge management system in the ED are shown in Figure 3 [9]. The figure shows that the *participants* use *information* and *technologies* in various *activities and processes* to create *products and services* to serve their *customers* (ie, *the 6 internal elements*). The *environment*, *strategies*, and available *infrastructure (*ie, *the 3 external elements)* also influence the work system. The arrows indicate that the specific elements in the work system must be in alignment.

We started by producing a list of codes (ie, the template) for each of the 9 elements of the WST and their interactions (ie, a code for participants, a code for activities and processes, and a code for the interaction between these elements). This enabled us to identify conceptual themes and then cluster them into broader groupings. Then, we created a hierarchical organization of codes, with groups of similar codes clustered together to produce more general high-order codes. For example, separate codes relating to different groups of *activities and processes* were incorporated into high-order activities and processes code. Then, this was further subdivided into codes to capture different activities and processes, and these were further divided into factors influencing when and how different activities and processes were followed, codes capturing the challenges with performing the different activities and processes, and codes capturing the interventions that the hospital implementation team conducted to deal with the challenges that arose (ie, changing the triage process in the ED, initiating training, or adding an extra physician). As we coded the transcripts and marked them with the appropriate code, we revised the template as needed. For example, as we identified an issue that was not covered by an existing code, we added a new code; we also deleted codes if we found that there was no need to use it. This was an iterative process of reading the transcripts, assigning codes, and reviewing the coding template until we were confident that the template was sufficiently clear and comprehensive.

**Figure 4:**
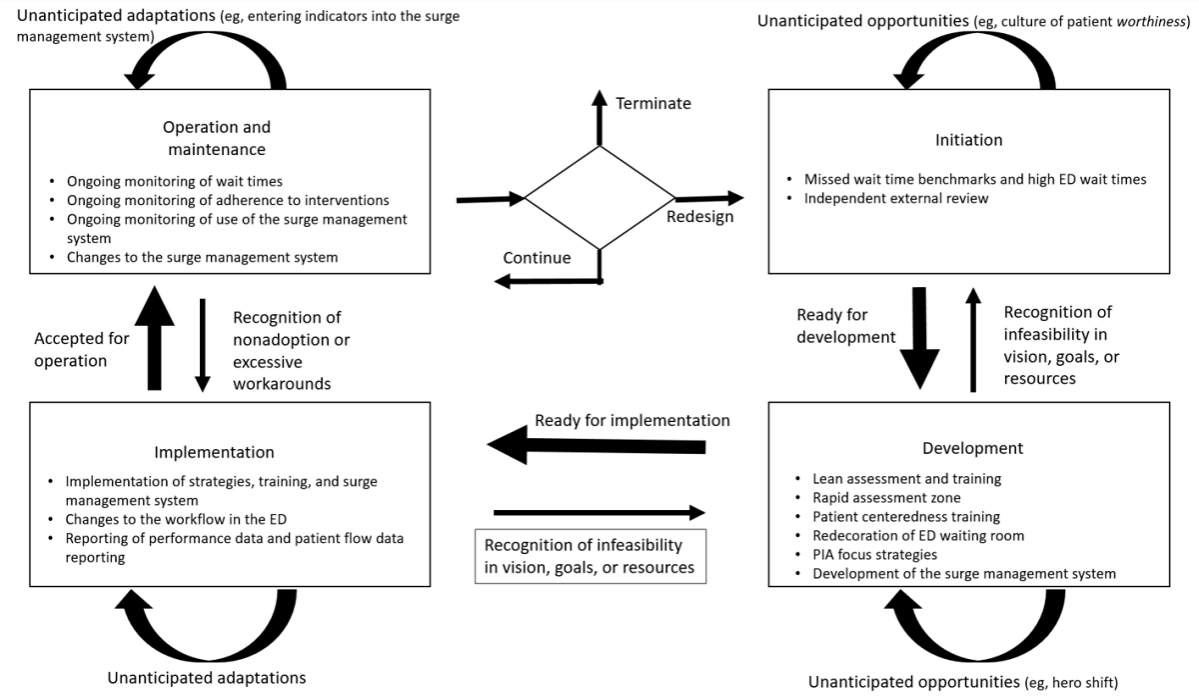
Work system life cycle model for the surge management system (adapted from the publication by Alter [9]). ED: emergency department; PIA: provider initial assessment.

Next, we used template analysis to code the WSLC model to capture the iterative process through which the system was implemented from initiation to operation and maintenance to identify planned and unplanned changes and the resulting interventions. Figure 4 [9] shows the WSLC model, adapted to the implementation of the surge management system. We coded the data according to the 4 phases of the WSLC model (ie, initiation, development, implementation, and operation and maintenance), interactions between the phases, planned and emergent changes, and outcomes. Similar to the coding of the static framework, this was an iterative process involving coding and further refinement of the template until we reached a state that enabled us to capture the WSLC model. Once all the transcripts were coded according to the final templates for the static framework and the WSLC model, we reviewed the coded text to identify themes and relationships between them.

Finally, we obtained data from the ED to measure wait times before, during, and after our study period. The data contained the patient ED wait times from patient registration to patient departure from the ED. We analyzed the data from approximately 1 year before our study started to 1 year after our study was completed (April 1, 2017, to March 31, 2021). We collected data for total patient visits, time to provider initial assessment (PIA), length of stay for departed patients (LOSDep), and patients who left without being seen. Time to PIA is the elapsed time from the point a patient first registers at the ED until the designated provider (ie, physician or nurse practitioner) makes contact. LOSDep is the time interval between a patient’s arrival to the ED to the time the patient physically leaves the ED. The number of patients who left without being seen is the percentage of patients who have registered at the ED and have been triaged but leave before being seen by a designated provider.

### Ethics Approval

Participants provided informed consent, and the Memorial University’s Interdisciplinary Committee on Ethics in Human Research approved the study (20190669-BA).

## Results

### Summary

The results indicate that WST is a useful approach to structure the implementation of an eHealth system because it incorporates the entire work system, and not just the eHealth system, in the implementation framework. In this section, we identify the role that specific elements of WST’s static framework and dynamic WSLC model played in the implementation and demonstrate how WST can be applied in the health care setting to guide the implementation of an eHealth system. Although the implementation team did not follow WST specifically, they included many of its elements in their approach.

### Static Framework

#### Overview

We found that the hospital implementation team began the project with an independent external review to assess the organization and function (ie, the elements) of the ED work system. Then, they designed interventions to address the issues that were found. They did not just examine the eHealth system portion of the surge management process, rather, they recognized the radical change that they were trying to implement in the ED and examined the entire work system. A member of the implementation team commented the following during the implementation of the surge management system:

I guess the electronic piece [of the surge management system] is one thing, but there’s also the strategy that goes along with it and we’re examining that in the broader sense. And the strategy is controversial, a little bit. So if you don’t have supportive buy-in from some of the frontline staff, and if it’s not presented in a way that would be well explained, it could be definitely seen as something that people wouldn’t want to do.

#### Internal Environment

The implementation team recognized the need to focus their efforts on meeting the needs of their *customers* (ie, the staff, hospital administration, and patients). They involved the staff in the implementation. A staff member commenting on the implementation of the surge management system said the following:

Management driven, it’s frontline driven from us. And we’re like, what can we change about this process.

The implementation team designed strategies to address their customers’ (ie, staff and patient) issues. For example, time to PIA is strongly correlated with patient satisfaction; thus, they developed strategies (ie, briefly assessed patients even when formal assessment space was not immediately available or emergency physicians could triage with nursing staff in the triage room without waiting and discharge if applicable) to reduce time to PIA. As can be seen in Figure 3 [9], this is a basic component of the WST static framework, which places customers at the top of the triangle. An aspect of the implementation that could have been improved in the beginning was staff training on the overall new surge management process. As we describe in the following sections, the implementation team realized that more training was needed during the implementation for staff entering data into the surge management system; however, their approach to training focused primarily on learning the job and did not always seem to provide the staff an understanding of the overall purpose of the surge management changes or their role in the process. There were comments that the way the surge management process functioned depended on who was on shift that day, and that, in particular, physicians can have a big impact on the process. In situations such as the ED, where teamwork is so important, and as one staff member commented, “You’re making decisions on the fly and you can’t really plan ahead,” a shared purpose and approach to surge management is needed. Furthermore, most survey respondents (11/18, 61%) indicated (agreed or strongly agreed) that they thought that the surge management system had improved their productivity and ability to coordinate continuity of care (11/18, 61%) and improved the hospital’s patient care delivery, productivity, and clinical outcomes (Table 2—user satisfaction and benefits to hospital); however, in the interviews, most staff indicated that they were not aware of the real impact on patients or efficiency in the ED. They had not seen the time to PIA, LOSDep, number of patients who left without being seen, or surge levels over time; thus, they were not sure what benefits were realized. A staff member commented the following:

It’d be nice to see the stats and if [the surge management system] was actually related in respect to getting patients to the floor. Like door-to-doctor, or triage-to-doctor, that kind of stuff...or what’s decreasing over time. That would be nice to see.

The implementation team did not include patients, and the project did not measure patient satisfaction. Following a WST approach may have helped to ensure that they had designed the implementation with a focus on customers (ie, staff and patients), and some of these issues may have been addressed at the onset of the project.

Despite some issues with implementation, many elements of the static framework were applied. We found that rather than just implementing the eHealth system portion of the surge management system (ie, the *technology*), the implementation team recognized that there was a need to change *activities and processes*, adjust *participant* perceptions of how the ED should function, and track and share various *information* such as performance indicators (Figure 3) [9]. The implementation team was redesigning the ED’s work system to address areas of misalignment identified between elements of the static framework. For example, when the implementation team recognized that waiting for beds for patient assessment, blood tests, electrocardiograms, and other minor procedures were contributing to overcrowding in the ED, they created a rapid assessment fast-track zone. When they recognized the need to decrease time to PIA, they designed changes in the workflow to reduce the time to PIA. These changes to the work processes helped to address the misalignment between the *activities and processes* and *products and services* elements and focus the work system on providing patient care and decreasing wait times. Interventions to address the misalignment between *participants* and *activities* *and processes* were addressed through lean training for frontline ED staff to encourage them to become active participants in the improvement process and redecoration of the waiting and examination rooms to have a more inviting environment for patients. Regular performance reporting facilitated alignment between *information* and *activities and processes*. The implementation team also used the information obtained from the external review and through the various interventions to design and develop a surge management system that was aligned with the activities and processes in the ED. The team recognized that the eHealth portion of the surge management system was only a component of the ED work system. They understood the impact and importance of the changes on other elements of the work system. A staff commented the following:

All of these different things, super track, nurse practitioner, the hero shift...some days we’d sink if we never had it.

**Table 2:** Staff perceptions of the surge management system as indicated in the survey.

			Value, mean (SD; range)
**User satisfaction^a^**			
	How satisfied are you with the surge management system?^b^	3.52 (1.08; 1-5)	
	The surge management system improves my productivity.	3.71 (1.10; 2-5)	
	The surge management system enhances my ability to coordinate continuity of care.	3.76 (1.18; 2-5)	
	The surge management system makes my job easier.	3.48 (1.12; 1-5)	
	The surge management system improves the quality of care that I can provide.	3.52 (1.12; 2-5)	
	The surge management system improves the quality of my decision-making.	3.48 (1.12; 1-5)	
**Benefits to hospital^a^**			
	Using the surge management system has improved patient care delivery.	3.94 (0.97; 1-5)	
	Using the surge management system has improved clinical outcomes.	3.82 (0.95; 2-5)	
	The surge management system improves our productivity.	4.12 (0.99; 1-5)	
**Job level change^a,c^**			
	I am expected to do more work than I used to.	2.78 (0.85; 2-5)	
	The nature of my work has changed.	3 (0.90; 2-5)	
	My job responsibilities have changed.	2.91 (1; 2-5)	
	I find greater demands placed on me at work because of this change.	2.91 (1; 2-5)	
	I am experiencing more pressure at work because of this change.	2.74 (1.01; 1-5)	
	The work processes and procedures I use have changed.	2.96 (0.88; 2-5)	
	My use of the surge management system is integrated with my workflow.	4.06 (0.83; 2-5)	
**Change fairness^a^**			
	Sufficient advanced notice was given to employees affected by the change.	3.96 (1.40; 1-5)	
	Those affected by the change had ample opportunities for input.	3.78 (1.35; 1-5)	
	The hospital kept everyone fully informed during the change.	3.65 (1.27; 2-5)	
	People affected negatively by this change were treated fairly.	3.78 (1; 2-5)	
**Management support for the change^a^**			
	Sufficient resources were available to support this change.	4 (0.85; 2-5)	
	All levels of management were committed to this change.	3.95 (0.84; 2-5)	
	Management dealt quickly and effectively with surprises during the change.	3.59 (0.91; 2-5)	
	There was sufficient management support for this change.	3.82 (0.96; 2-5)	
	Management was supportive of this change.	4.05 (0.67; 3-5)	
	People in this hospital find their work more interesting.	3.32 (1.13; 1-5)	
	Most people in this hospital are better off.	3.35 (1.19; 1-5)	
	People’s quality of life at work has improved.	3.36 (1.14; 2-5)	
**Commitment to change^a^**			
	This change serves an important purpose.	4.04 (0.82; 2-5)	
	I believe in the value of this change.	4 (0.90; 2-5)	
	This change is a good strategy for this organization.	4.09 (0.73; 3-5)	
	I think management is making a mistake by introducing this change.^c^	1.91 (0.79; 1-4)	
	Things would be better without this change.^c^	1.83 (0.72; 1-3)	
	This change is not necessary.^c^	1.78 (0.74; 1-3)	

^a^Scoring: 1=strongly disagree, 2=disagree, 3=neutral, 4=agree, and 5=strongly agree.

^b^Scoring: 1=extremely dissatisfied, 2=dissatisfied, 3=neither satisfied nor dissatisfied, 4=satisfied, and 5=extremely satisfied.

^c^Reverse score.

#### External Environment

According to WST, the 6 elements of the static framework are influenced by the 3 elements of the external environment of the hospital that affect the work system (Figure 3 [9])—the *environment*, *strategies*, and *infrastructure*. First, within the environment, the culture had a major impact on the implementation of the system. The ED staff had a history of resistance to change and certain beliefs about who should be in the ED. Commenting on staff acceptance of the changes related to the surge management system, a staff member said, “I think it was really hard to go from being very rule oriented to being more flexible.” The implementation team created patient-centeredness training to address the belief system that patients with low acuity should not seek care in the ED. One of the members of the implementation team commented, “We changed a complex system of beliefs.” Other factors such as established processes and professional practices also created some resistance to the new approach. A member of the implementation team commented the following:

We have been, since 1998 in Canada, seeing patients based on Canadian triage acuity scale. [The surge management system] kind of meddles with that a little bit.

These initiatives appeared to bring staff on board and create a culture that was more accepting of change. In addition, a change committee was established to help communicate and identify needs for change on an ongoing basis. Second, the *strategies* at the hospital, ED, and work system were in alignment. The health authority, hospital, and ED were focused on improving patient care in the ED and supportive of innovative approaches. A manager commented the following:

[The health authority] has tried to put a more innovative angle in healthcare and [this hospital] been chosen as there are innovative kind of people in the frontline doing some things that can help get innovation into healthcare. Another strategy is probably improving patient satisfaction...We know that usually what happens in the ED is often reflected in how well your hospital works and how well patients are satisfied. If you don’t have a good ED, you're not going to be reflected as having a good hospital. So the more that we get things improved in the ED, it tends to transform and cross over to other hospitals or areas.

The hospital management and ED management were in support of this initiative and devoted staff and funds to support the project, and their strategies were aligned and focused on reducing ED wait times. Third, there were some issues with the infrastructure as they were not able to connect the eHealth portion of the surge management system with the hospital’s other systems; therefore, staff had to continue to input the values for the indicators into the surge management system. There appeared to be some confusion regarding the responsibility for this task, and we observed that sometimes, the indicators were not entered regularly. This impaired the use of and possible benefits from the system.

#### Outcomes of the Implementation

The strength of this whole system approach to structuring the implementation is reflected in the success of the surge management system. The new approach to surge management changed the work system in the ED (Table 2—job level change), with comments from staff such as, “It’s a whole new way of thinking.” Despite these changes in workflow in the ED, we observed a strong commitment to the system (Table 2—user satisfaction). In total, 72% (13/18) of the survey participants were satisfied or extremely satisfied with their use of the system, 82% (14/17) would like to increase or significantly increase their use of the system in the future, and 82% (14/17) agreed or strongly agreed that it was integrated with their workflow.

We observed that most staff accepted the new collaborative approach to surge management (eg, “People are willing to work together and help”), and many commented that the patients seemed happy (eg, “A lot of people seem happier”). Furthermore, participants indicated that they agreed or strongly agreed that the system has improved patient care delivery (14/17, 82%), clinical outcomes (10/17, 59%), and productivity (15/17, 88%; Table 2—benefits to hospital).

**Figure 5:**
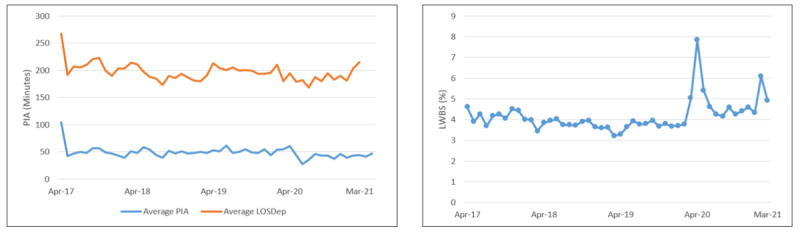
Average time to provider initial assessment (PIA), length of stay for departed patients (LOSDep), and patients who left without being seen.

The ED wait time data indicated an improvement in ED wait times at the beginning of the implementation from October 1, 2014, to March 31, 2017. Despite approximately 26% increase in patient volume, the time to PIA decreased by 62.1 minutes, LOSDep decreased by 65 minutes, and patients who left without being seen decreased from 12.1% to 4.6% [21]. Our study began approximately 1 year later when the eHealth system portion of the surge management system was being implemented. Our analysis shows that the wait times have plateaued, but stayed consistent since the time of initial implementation and through the implementation of the eHealth system portion of the surge management system, despite the increase in patient volume. Table 3 shows the characteristics of patient visits to the ED from April 1, 2017, to March 31, 2021. The dramatic decrease in ED wait times after the initial implementation of the surge management system and the ability of the ED to maintain these wait times in the 4 years after the implementation and through the implementation of the eHealth system portion of the surge management system can be seen in Figure 5. Slight variations in the time to PIA and LOSDep and large increase in the number of patients who left without being seen can be seen around the beginning of the COVID-19 pandemic in March 2020; however, since this time, the wait times have returned to their prepandemic values.

**Table 3:** Characteristics of patient visits to the emergency department (2013-2021).

Characteristics	July 1, 2013, to September 30, 2014^a^	January 1, 2016, to March 31, 2017^a^	April 1, 2017, to March 31, 2018	April 1, 2018, to March 31, 2019	April 1, 2019, to March 31, 2020	April 1, 2020, to March 31, 2021
Total visits, n	23,898	30,031	26,966	29,321	29,014	22,931
Number of daily visits, mean (SD)	52^b^	66^b^	74 (6.54)	80 (6.25)	79 (8.03)	63 (10.05)
Time to PIA^c^ (minutes), mean (SD)	104.3 (0.9)	42.2 (8.1)	49.6 (5.5)	48.5 (4)	51.9 (5.7)	41.3 (5.5)
LOSDep^d^ (minutes), mean (SD)	199.4 (16.8)	134.4 (14.5)	158.2 (7.3)	139.7 (8.3)	147.8 (10)	145.6 (11.5)
Patients who left without being seen (%), mean (SD)	12.1 (2.2)	4.6 (1.7)	4.1 (0.3)	3.7 (0.2)	3.8 (0.4)	5.0 (1.1)

^a^These data were obtained from the publication by Patey et al [21].

^b^SD value is unavailable.

^c^PIA: provider initial assessment.

^d^LOSDep: length of stay for departed patients.

### WSLC Model

In contrast to the static framework that we used to examine the work system at one point in time, we applied the WSLC model to examine the changes to the work system over time through planned and unplanned changes as part of the system’s natural evolution. We found that the surge management system project followed the 4 phases of the WSLC: initiation, development, implementation, and operation and maintenance (Figure 4) [9]. The project was initiated to address missed wait time benchmarks and high ED wait times, and it was initiated with the independent external review. As we discussed, this review identified areas for improvement, and interventions for a new surge management system were created in the development phase, planned strategies were implemented in the ED, and surge management system process and outcomes were continuously monitored. Initially, continuous monitoring was performed informally; however, as we mentioned previously, a change committee was created to take a more systematic approach to monitor the surge management system and make the necessary changes over time.

The planned changes included the eHealth system portion of the surge management system itself, along with the interventions described previously; however, in addition, we found that unanticipated opportunities and challenges emerged through the development cycle. For example, during initiation, the implementation team found that in addition to the changes in workflow, training, and creation of the surge management system that the team thought they would have to make, they discovered that they also needed to create a culture of patient *worthiness*, and an intervention to address this change was developed. Throughout the phases of the project, we found that the implementation team followed an iterative approach. When they recognized that changes were necessary in the static framework elements, they had the authority to make those changes. An implementation team member commented the following:

It’s interim improvements as we go...where we’re going to fix what we see is wrong before we go anywhere else.

During the implementation phase, the team found that during the busy times, despite following the new surge management policies and procedures, staff were still often unable to manage the wait times sufficiently. Therefore, the team went back to the development phase and created a new *hero* shift to provide added ED physician and nursing support during these times. This was an unanticipated opportunity and proved to have beneficial results. In addition, during the operation and maintenance phase, the team saw unanticipated adaptations in the frequency with which staff entered the surge measures in the surge management system, and additional staff training was conducted. The implementation team also saw issues with accessing information for some indicators, and the surge management system was modified accordingly.

We found that the iterative approach helped the team to address issues as they arose. However, the changes required by the new ED surge management process were not easily made, and the implementation team faced some resistance. Staff commented the following:

Some people understand the process of flow better than others.

And then you’ve got some doctors, it’s like pulling teeth...they’re just associated with the old way of doing things.

I think it was really hard to go from being very rule-oriented to being more flexible.

The implementation team recognized the challenges with the new system:

The strategy is controversial...it could be definitely seen as something that people wouldn’t want to do.

For example, the changes included broadening the scope of work for the primary care paramedics to include transporting patients to an admitting floor. This could have been perceived as negative, but survey participants indicated a high degree of change fairness (Table 2):

People affected negatively by this change were treated fairly.

The implementation team commented that the challenges to incorporating the surge management system into the daily workflow were exacerbated by the fact that they could not make the use of the system mandatory:

It’s really tough to do mandatory things. It’s not like a factory...most people got practices that are guided by professional bodies...probably the biggest challenge here is that.

However, we found that, overall, the staff indicated that the changes were managed well, as demonstrated by agreement levels (Table 2—change fairness, management support for the change, and commitment to change). Sufficient resources were available to support the change (14/20, 70% agreed or strongly agreed), there was sufficient management support for the changes (15/19, 79%), and the change served an important purpose (16/20, 80%).

The need for an iterative approach was also reflected in the fact that the ED work system itself was not stable. We saw that there were changes to the surge management system, new staff, new activities and process, and other new systems. An ED staff member commented the following:

...Change is now part of regular work...and here it seems to just be part of what you do now. It’s actually become just regular work.

This reflects the need for continuous monitoring of the surge management process and illustrates how the WSLC model can be used to manage the implementation to recognize unanticipated opportunities and adaptations over time.

## Discussion

### Principal Findings

The broad scope and holistic approach of WST is well suited to guide eHealth system implementations in the ED as it has the potential to address a key barrier to successful implementations—integrating the system into clinicians’ work. EDs are complex systems that involve a variety of groups responsible for guiding patients through different organizational and clinical processes during their care. System implementation in such a complex setting requires focus on the work system, and not just the IT artifact. The surge management system is an example of a successful implementation, as the surge management system and related interventions improved key ED wait times. In our study, we demonstrate what made this implementation a success, through a WST analysis lens. It is important to understand what aspects of the implementation made it a success because if we understand what works, other implementation teams also can use this approach. We found that the key success factors were the incorporation of the entire work system into the implementation framework and the iterative approach.

Although the implementation team did not follow WST, we propose that it can be applied in the implementation of eHealth systems in such environments. The WST’s static framework can help to broaden the focus of implementation from just the eHealth system to a view that considers the eHealth system as a part of a large work system, in which human and technological components work together to manage patient care. The emphasis on the services produced and the value of those services to the staff and patients is particularly beneficial in the health care setting. This will ensure that the implementation is focused on the end user and the ultimate goal of the system, rather than a narrow focus such as system’s use. We saw that the external review helped to identify all the elements of the ED work system and not only the eHealth system portion of the surge management system that can affect the success of the system implementation. The resulting interventions helped to ensure alignment among elements in the WST’s static framework and to focus the attention throughout implementation on the ultimate goal of reducing ED wait times.

We saw how taking a more holistic approach for the implementation helped to alleviate some of the common barriers to eHealth system success. For example, rather than focusing on the technical components of the surge management system, something that is a significant contributor to the gap between prospect and reality [22,23] and which is only a component of the WST static framework, the implementation team also focused on the other elements of the static framework. Furthermore, they addressed resistance to change, which is another barrier to eHealth interventions [24,25], by involving staff in the implementation, through training and creation of a change committee. We saw how they considered the different perspectives of different staff and management involved in the surge management process by involving them in the implementation process, something, which if not done can be another barrier [26,27]. There is a widespread perception in human-computer interaction that recognizes the importance of user-centered design and participatory design approaches. This has also been shown to be important in the design of workflow associated with eHealth system implementations. On the basis of their review of the factors influencing the outcome of eHealth interventions, Granja et al [6] proposed that user involvement in the design of the workflow is the most important factor for the success of eHealth systems. Using the WST lens helped us to identify the different elements of the work system and discover how the interventions addressed any misalignment between the elements. We propose that applying the static framework will help implementation teams to ensure that they are including the essential elements of the work system and provide them with a systematic way to assess the alignment between the elements.

Viewing the implementation of the surge management system through the WSLC model created an approach that differs fundamentally from the traditional systems development life cycle. In the systems development life cycle methodology, the system is the technical artifact that is created, and it does not necessarily incorporate iterations. We found that viewing the surge management system as part of dynamic work processes with a series of changes that emerged through planned and unplanned events, and their interactions, highlighted the importance of continuously identifying the changes or areas of misalignment in the work system. It also helped to clarify what interventions were needed and how to manage such changes, while recognizing that changes to the work system can occur through planned initiatives or emerge over time. We propose that implementation teams can use the WSLC model to follow an iterative approach that will allow them to be open to unanticipated opportunities and recognize unanticipated adaptations during the implementation and give them a structured way of dealing with the planned and emergent changes.

Researchers have adapted and used WST in different areas over several decades [9,10]; however, it has had limited application in health care [28,29]. To the best of our knowledge, it has not yet been applied in the ED, nor has it focused on how it can be used during the implementation of an eHealth system. Therefore, we broaden the focus of WST by applying it in the health care field (specifically ED) and to its use in the implementation of eHealth systems. We propose that the static framework can be used by non-IT and IT professionals to analyze the work system to incorporate the sociotechnical aspects of implementing an eHealth system as part of a large work system. Then, the WSLC model can be used to structure the implementation and monitor and manage the system over time. Future studies can examine the implementation of an eHealth system with a team that is following the WST approach, to uncover any problems that may arise and identify opportunities for overcoming them.

### Limitations

This study was conducted in 1 rural hospital ED. Experiences regarding the implementation of the same or other ED systems may be different in other types of ED settings, thus limiting generalizability. However, the practices of WST have been shown to improve implementation success in other complex settings. Another limitation is the small sample size of the survey; however, approximately half of the ED staff responded. Moreover, the observations were conducted only for 2 days; however, during observation, we asked if the shifts represented a typical day, and they indicated that they did. They also indicated that after 10 PM, the ED is generally not busy, and thus, surge management during nights is similar to a nonbusy time during the day. The ED wait time data before, during, and after implementation of the surge management system support the success of this implementation. The use of the mixed methods approach with surveys, interviews, observations, document review, and ED wait time data analysis should have helped to combat potential limitations.

### Conclusions

This conceptualization of the surge management system implementation through the lens of WST gives us insight into how to structure the implementation of a surge management system to incorporate the broad work system. We captured how the static framework can be a useful tool to assess the elements of the broad work system that need to be changed and managed to successfully implement an ED surge management system, and we propose that the WSLC model can provide a structured way to manage the implementation of such changes. This study addresses the need for more studies on surge management systems and methodologies to implement eHealth systems that incorporate the broad work system. These findings can guide further studies and implementations of eHealth systems.

## Appendix

Multimedia Appendix 1 Survey questions, interview questions, and observation protocol.

## Abbreviations

ED: emergency department
LOSDep: length of stay for departed patients
PIA: provider initial assessment
WSLC: work system life cycle
WST: work system theory

## References

[1] Morley C, Unwin M, Peterson GM, Stankovich J, Kinsman L (2018). Emergency department crowding: a systematic review of causes, consequences and solutions. PLoS One.

[2] Saghaeiannejad-Isfahani S, Hazhir F, Jalali R (2019). An assessment of emergency department information systems based on the HL7 functional profile. J Educ Health Promot.

[3] Callen J, Li L, Georgiou A, Paoloni R, Gibson K, Li J, Stewart M, Braithwaite J, Westbrook JI (2014). Does an integrated Emergency Department Information System change the sequence of clinical work? A mixed-method cross-site study. Int J Med Inform.

[4] Gagnon M, Desmartis M, Labrecque M, Car J, Pagliari C, Pluye P, Frémont P, Gagnon J, Tremblay N, Légaré F (2012). Systematic review of factors influencing the adoption of information and communication technologies by healthcare professionals. J Med Syst.

[5] Ross J, Stevenson F, Lau R, Murray E (2016). Factors that influence the implementation of e-health: a systematic review of systematic reviews (an update). Implement Sci.

[6] Granja C, Janssen W, Johansen MA (2018). Factors determining the success and failure of eHealth interventions: systematic review of the literature. J Med Internet Res.

[7] Kawamoto K, Houlihan CA, Balas EA, Lobach DF (2005). Improving clinical practice using clinical decision support systems: a systematic review of trials to identify features critical to success. BMJ.

[8] Kruse CS, Regier V, Rheinboldt KT (2014). Barriers over time to full implementation of health information exchange in the United States. JMIR Med Inform.

[9] Alter S (2013). Work system theory: overview of core concepts, extensions, and challenges for the future. J Assoc Inform Syst.

[10] Alter S (2015). Work system theory as a platform: response to a research perspective article by Niederman and March. J Assoc Inf Syst.

[11] Allaudeen N, Vashi A, Breckenridge JS, Haji-Sheikhi F, Wagner S, Posley KA, Asch SM (2017). Using lean management to reduce emergency department length of stay for medicine admissions. Qual Manag Health Care.

[12] Arbune A, Wackerbarth S, Allison P, Conigliaro J (2017). Improvement through small cycles of change: lessons from an academic medical center emergency department. J Healthc Qual.

[13] Aksel G, Bildik F, Demircan A, Keles A, Kilicaslan I, Guler S, Corbacioglu SK, Turkay A, Bekgoz B, Dogan NO (2014). Effects of fast-track in a university emergency department through the National Emergency Department Overcrowding Study. J Pak Med Assoc.

[14] (2015). System and use assessment survey. Canada Health Infoway.

[15] Caldwell SD, Herold DM, Fedor DB (2004). Toward an understanding of the relationships among organizational change, individual differences, and changes in person-environment fit: a cross-level study. J Appl Psychol.

[16] FEDOR DB, CALDWELL S, HEROLD DM (2006). The effects of organizational changes on employee commitment: a multilevel investigation. Pers Psychol.

[17] Herscovitch L, Meyer JP (2002). Commitment to organizational change: extension of a three-component model. J Appl Psychol.

[18] William HD, Ephraim RM (2014). The DeLone and McLean model of information systems success: a ten-year update. J Manag Inform Syst.

[19] Glaser BG, Strauss AL (1967). The Discovery of Grounded Theory Strategies for Qualitative Research.

[20] King N (2004). Using templates in the thematic analysis of text. Essential Guide to Qualitative Methods in Organizational Research.

[21] Patey C, Norman P, Araee M, Asghari S, Heeley T, Boyd S, Hurley O, Aubrey-Bassler K (2019). SurgeCon: priming a community emergency department for patient flow management. West J Emerg Med.

[22] Biggs K, Lowe P, Walsh J, Lagios K (2010). Audit of a sexual health website email link for general practitioners. Int J STD AIDS.

[23] Declerck G, Aimé X (2014). Reasons (not) to spend a few billions more on EHRs: how human factors research can help. Yearb Med Inform.

[24] LeTourneau B (2004). Managing physician resistance to change. J Healthc Manag.

[25] Narine L, Persaud DD (2003). Gaining and maintaining commitment to large-scale change in healthcare organizations. Health Serv Manage Res.

[26] Kapadia V, Ariani A, Li J, Ray PK (2015). Emerging ICT implementation issues in aged care. Int J Med Inform.

[27] Frauenberger C, Good J, Fitzpatrick G, Iversen OS (2015). In pursuit of rigour and accountability in participatory design. Int J Hum Comput Stud.

[28] Dadgar M, Joshi K (2018). The role of information and communication technology in self-management of chronic diseases: an empirical investigation through value sensitive design. J Assoc Inf Syst.

[29] Johnsen HM, Fruhling A, Fossum M (2016). An analysis of the work system framework for examining information exchange in a healthcare setting. Commun Assoc Inf Syst.

