# Supplementary material for: Investigating a Work System Approach to Implement an Emergency Department Surge Management System: Case Study
Source: J Med Internet Res. 2022 Aug 25;24(8):e37472. doi: 10.2196/37472 (PMC9459829; doi:10.2196/37472)
Supplement: Multimedia Appendix 1 [file jmir_v24i8e37472_app1.docx]

# Multimedia Appendix

## Survey Questions

Demographic questions: current role at the hospital, gender, age, describe your use of the surge management process, how long have you been using the new surge management process or been impacted by its use, typical work shift, how long have you worked at this hospital.

|  |
| --- |
| *User satisfaction^a^* |
| How satisfied are you with the surge management system?^b^ |
| The surge management system improves my productivity |
| The surge management system enhances my ability to coordinate continuity of care |
| The surge management system makes my job easier |
| The surge management system improves the quality of care that I can provide |
| The surge management system improves the quality of my decision making |
| *Benefits to hospital^a^* |
| Using the surge management system has improved patient care delivery |
| Using the surge management system has improved clinical outcomes |
| The system improves our productivity |
| *Job level change^a,c^* |
| I am expected to do more work than I used to |
| The nature of my work has changed |
| My job responsibilities have changed |
| I find greater demands placed on me at work because of this change |
| I am experiencing more pressure at work because of this change |
| The work processes and procedures I use have changed |
| My use of the surge management system is integrated with my workflow. |
| *Change fairness^a^* |
| Sufficient advanced notice was given to employees affected by the change |
| Those affected by the change had ample opportunities for input |
| The hospital kept everyone fully informed during the change |
| People affected negatively by this change were treated fairly |
| *Management support for the change^a^* |
| Sufficient resources were available to support this change |
| All levels of management were committed to this change |
| Management dealt quickly and effectively with surprises during the change |
| There was sufficient management support for this change |
| Management was supportive of this change |
| People in this hospital find their work more interesting |
| Most people in this hospital are better off |
| People's quality of life at work has improved |
| *Commitment to Change^a^* |
| This change serves an important purpose |
| I believe in the value of this change |
| This change is a good strategy for this organization |
| I think management is making a mistake by introducing this change^c^ |
| Things would be better without this change^c^ |
| This change is not necessary^c^ |
| *^a^Scoring: 1=strongly disagree, 2=disagree, 3=neutral, 4=agree, and 5=strongly agree.*  *^b^Scoring: 1=extremely dissatisfied, 2=dissatisfied, 3=neither satisfied nor dissatisfied, 4=satisfied, and 5=extremely satisfied.*  *^c^Reverse score.* |

## Interview Questions

*Please note that the questions were adjusted depending on which stage of the implementation they were currently in when the interview took place.*

Demographic information: gender, age, role in the ED, involvement in the implementation of the new surge management process (including the system).

Questions for management:

- Why did the hospital decide to create the new surge management system?
- What is the normal patient volume/wait times? How many times do you reach the levels 3-5?
- Do the objectives of surge management system align with the strategic goals of the hospital? Please explain.
- Describe the implementation of the surge management system at the hospital (i.e. over a period of months, phases, etc.).
- What interventions were implemented at the hospital as part of this new surge management initiative?
- What planning, activities and resources were in place for the implementation and use of the surge management system? Communication, management commitment, training, monitoring, reporting, etc.
- What types of individuals/groups (i.e. clinicians, administrators, patients, others) have affected the implementation of the surge management system? How?
- How are decisions controlled in the emergency department (e.g. hierarchical, rigidly controlled vs staff can make changes)?
- What is the general attitude around change in the hospital? Anything else going on at the same time? (any other changes?)
- How has the culture of the hospital influenced the use of the surge management system? Do you anticipate any changes once start using the e-health system?
- What do you see as the differences with implementing such changes in the ED compared to other parts of the hospital or outside of the hospital setting?
- How would you describe the management support of this initiative?
- Did roles and responsibilities change (i.e. champions and staff participation) How was that handled? Training, documentation?
- How are different groups involved in the new surge management process? Will this change with the implementation of the e-health system?
- Did communication between management and the ED clinical staff change with the implementation of the surge management system?
- Do you see any impact of social influence on use of the surge management system?
- How did health information system standards influence the use and/or success of the surge management system?
- How did performance standards (i.e. for accreditation of the hospital and performance targets) influence the use and/or success of the surge management system?
- How did practice standards (i.e. professional competency) influence the use and/or success of the surge management system?
- What were/are the general expectations on the return on value from adoption of a health information system and surge management system in particular?
- What are the individual benefits (for different groups) and organizational benefits? How do individual benefits lead to organizational benefits?
- Anything unexpected happen? Counterintuitive? Influenced anyone else to make mistakes?
- Were expected individual benefits identified by the hospital?
- How did incentive programs entice change at the individual, practice and organizational levels influence use and/or success of the surge management system?
- How are employees evaluated? (i.e. were they evaluated on work processes or outcomes or anything else? Did they change?) How is ED or other departments evaluated? Has this changed?
- How did related legislative acts (i.e. privacy) influence use and/or success of the new surge management system?
- How did related regulations/policies (i.e. security or privacy guidelines) influence use and/or success of the surge management system?
- What is the accountability and decision-making structure regarding adopting the surge management system? How did they influence use and/or success of the surge management system?
- How did general expectations of the public toward healthcare influence use and/or success of the surge management system?
- How did general economic trends influence use and/or success of the surge management system?

Questions for clinicians involved in the surge management process:

- Based on your experiences to date with the surge management system, how acceptable is the quality of the system itself?
- How has the use of the surge management system changed your work processes?
- Does use of the surge management system fit with your work responsibilities (i.e. workflow)? If not, please explain. How has this impacted your use of the surge management system? Changed what you do? For the good? Bad?
- How did you used to know what surge procedures to follow, versus now with the surge management system? What will change with the e-health system portion?
- Has the use of the surge management system changed how you interact with others in your duties (i.e. shared responsibility)? Who do you interact with?
- Does your use of the surge management system change as the ED is busier?
- Has your use of the surge management system changed with the implementation of the e-health system portion as compared to the surge management system manual protocol?
- Do you anticipate any changes with use of the e-health portion of the surge management system?
- In general, when thinking about the quality of the information provided by the surge management system, how acceptable do you find the quality of the information to be (i.e. completeness, accurate, available, and relevant)?
- Information used to calculate the level is complete, info needed to calculate the level is quickly provided, level is accurate, relevant, and available when I need it, format and layout of protocols are acceptable. Please explain.
- In general, when thinking about the quality of the services (i.e. technical support and training services, implementation process, ongoing support, etc.) provided for the surge management system, how acceptable do you find the quality of these services to be? Please explain.
- How do you think using the surge management system will impact your job performance?
- Do you anticipate any changes with use of the e-health portion of the surge management system?
- Do you think the interaction with the surge management system is clear and understandable? Do you have the skills necessary? How did you get them?
- Who influences you most in your day-to-day work? How do these people feel about you using the surge management system? Have they encouraged you to use it?
- Have you seen these people using the surge management system and has it encouraged you to use it? Has it helped you to learn how to use it?
- How has the culture of the hospital influenced your use of the surge management system?
- In general, how satisfied are you overall with the surge management system?
- How comfortable are you following the surge management system protocols?
- Are there aspects of the surge management system that you would change, and if so, which ones would they be? Please describe your comments.
- Are you getting the performance benefits out of using the surge management system?
- Has training influenced your ability to use the surge management system?
- Can you comment on the way the changes associated with the surge management system were managed?
- Are you committed to this change?
- Do you anticipate any changes with use of the e-health portion of the surge management system?
- Given a choice, would you like to increase or decrease your future use of the surge management system?
- Likely to recommend the surge management system to other healthcare providers at other hospitals?
- What benefits have you seen from the use of the surge management system to your role in the ED compared to the old method (improve productivity)? How long did it take for these benefits to be realized?
- What benefits have you seen from the use of the surge management system to patients (improved clinical outcomes), hospital compared to the old method? How long did it take for these benefits to be realized?
- Are there any benefits that you feel will occur but that just haven’t happened yet? Why do you think they haven’t happened yet?
- Have there been any unanticipated outcomes of the surge management system? Please explain. What, if anything, was done to deal with these outcomes?
- Has there been any resistance to using the surge management system? If so, please describe. What was done to overcome this resistance? Was it effective?
- Are there any other benefits that you would like to discuss? Please explain.
- Do you anticipate any changes with use of the e-health portion of the surge management system?

Follow-up Questions (after the e-health portion of the surge management system was implemented):

- How has the surge management system changed over the past X months? Please explain.
- Has your use of the surge management system changed over time? Why or why not?
- Has anything changed in the organization, your role, etc. that has affected the surge management system use or success?
- Reflect back on the current practices, were they planned, what is different? Why?
- As you continue to use the surge management system, how has your use changed? Why?
- Are the current work routines as planned or did changes emerge from the work practices? Please explain. If they emerged please explain how that happened (i.e. accommodations to contingencies, breakdowns, exceptions, opportunities and unintended consequences). Why?
- How has management responded to gaps in use (system maintenance, executive commitment) (i.e. feedback from organization level to individual use)? How were individual users impacted by this?

## Observation Protocol

- Date, time, and place of observation
- Unstructured observation in the ED. As part of this activity, note down what is happening in the location including:
  - What is happening within the ED, what are they trying to accomplish
  - Describe layout of ED
  - Ambience, atmosphere, environment
  - Physical characteristics of the setting
  - How I am feeling and how I think this might influence what I am observing and recording
  - Roles in the ED, where they sit, who does what, etc.
  - Specific words, phrases, summaries of conversations, and insider language
- Probing questions during the observation: Talk aloud example, “Can you please talk aloud as you manage surge so that I can get a better understanding of what you are doing?”
- Participants were asked the following question to determine if the shift observed represented a typical day? Did your management of surge represent your typical shift? If not, please explain how it was different.
- Questions about people or behaviors at the site for future investigation.
- Ask analytic questions: What do I see going on here? What did I learn from these notes? Why did I include them?
